# Mixed-effects Cox models of alcohol dependence in extended families

**DOI:** 10.1186/1471-2156-6-S1-S127

**Published:** 2005-12-30

**Authors:** Jing hua Zhao

**Affiliations:** 1 Department of Epidemiology & Public Health, University College London, 1-19 Torrington Place, London WC1E 6BT, UK

## Abstract

The presence of disease is commonly used in genetic studies; however, the time to onset often provides additional information. To apply the popular Cox model for such data, it is desirable to consider the familial correlation, which involves kinship or identity by descent (IBD) information between family members. Recently, such a framework has been developed and implemented in a UNIX-based S-PLUS package called *kinship*, extending the Cox model with mixed effects and familial relationship. The model is of great potential in joint analysis of family data with genetic and environmental factors. We apply this framework to data from the Collaborative Study on the Genetics of Alcoholism data as part of Genetic Analysis Workshop 14. We use the S-PLUS package, ported into the R environment , for the analysis of microsatellite data on chromosomes 4 and 7. In these analyses, IBD information at those markers is used in addition to the basic Cox model with mixed effects, which provides estimates of the relative contribution of specific genetic markers. D4S1645 had the largest variance and contribution to the log-likelihood on chromosome 4, but the significance of this finding requires further investigation.

## Background

While most genetic data analyses focus on disease events, ages of disease onset contain valuable information. Software for such analyses has been limited, and include the AGEON module in SAGE, the LINKAGE package that allows age classes with different penetrances in these classes, and the computer program LIPED that allows a log-normal/straight-line distribution. The Cox model for survival analysis is well established and has been extended to include random effects. When applied to family data, it is necessary to account for familial correlation and to include the identity-by-descent (IBD) information. Among models recently proposed with these component(s) [1-4], the framework by Therneau [2] appears to be the most comprehensive. Building on the established module *survival *in S-PLUS, a new UNIX package *kinship *has been developed, which contains, among others, a function called *coxme *that includes components of the Cox model, random effects, and familial relationship.

Earlier reports on candidate genes for alcohol dependence [5] showed loci on several chromosomes including 4 and 7, but were limited in the use of age-of-onset information and confounding factors. Here, the new modelling framework is explored with Genetic Analysis Workshop 14 (GAW14) problem 1 data using microsatellite markers on chromosomes 4 and 7.

## Methods

### The mathematical model

The mathematical model can be sketched as

Cox model + random effects = kinship/IBD information,

which is an extension of the standard Cox model for event-times to allow for random effects and the kinship or IBD information in families.

Following Ripatti and Palmgren [6], let *T*_*i *_denote the event time for unit *i*, *i *= 1, ..., *n*, *C*_*i *_the censoring time, *U*_*i *_= min(*T*_*i*_,*C*_*i*_), and *δ*_*i *_= *I*(*T*_*i *_≤ *C*_*i*_), the basic Cox model with vector of explanatory variables *X*_*i *_is specified via a hazard function *λ*_*i*_(*t*) = *λ*_0_(*t*)exp(*X*_*i*_*β*).

The model can be extended to include random effects or frailties *Z*_*i*_, such that *λ*_*i*_(*t*|*b*) = *λ*_0_(*t*)exp(*X*_*i*_*β *+ *Z*_*i*_*b*), b ~ *p*(*b*;*D*(*θ*)), with *θ *being a vector of unknown parameters. The likelihood function is similar in form to that of the partial likelihood of the standard Cox model. If the censoring is independent and non-informative of *b*, the likelihood function *L *in terms of (*λ*_*0*_(*t*),*β*,*θ*) can be obtained by integrating over *b*. Assuming *b *~ *MVN*(*b*,*D*(*θ*)) and defining  as usual, we have



Next, a Laplace approximation is applied to obtain an approximate marginal log-likelihood that can be maximized by a penalized fixed-effects partial likelihood for parameters (*β*(*θ*),*b*(*θ*)) and in turn used in a profile likelihood function involving only *θ *[6]. Here the parameters of interest *θ *are the variances involving kinship () and IBD () matrices, such that *D*(*θ*) is a linear combination of *θ *and these matrices. This is reminiscent of a similar approach found in SAS PROC MIXED, and the specific relationship between the Newton-Raphson iteration of a Cox model and a linear mixed-effects model has been elaborated elsewhere [7]. Further mathematical details, including the integrated and penalized likelihood and their degree(s) of freedom, together with an S-PLUS package for UNIX called *kinship *by Terry Therneau are available from . The specific function *coxme *generalizes *coxph *in the widely used S-PLUS package *survival *by the same author. Due to some difficulties with the S-PLUS package, it was ported into the R system . It takes advantage of the recent developments in S3/S4 classes [8] and is freely available.

### The data

The GAW14 problem 1 contains data from the Collaborative Study on the Genetics of Alcoholism (COGA), including a large number of pedigrees, microsatellite markers, and SNPs. A number of phenotypes and covariates are also available. The alcoholism diagnoses were based on DSM-III-R, Feighner, and DSM-IV criteria with information on ages of onset.

All 143 families in the GAW14 COGA data were used. These families had an average of 11 members (range 5 to 32) and the total sample size was 1,614 (826 men, 788 women). Two variables represented definitions of alcoholism: ALDX1 according to DSM-III-R + Feighner definition, and ALDX2 based on DSM-IV. ALDX1 and ALDX2 largely agree but ALDX1 is less stringent, with 103 "unaffected with some symptoms" under ALDX2 classified as affected under ALDX1. According to ALDX1, there were an average of 3 affected members (range 1 to 14) in these families. In the following analysis, ALDX1 will be used. Possible confounding variables include sex, ethnicity, and smoking. We did not adjust for ethnicity in the analysis. The raw data were extracted and analyzed using C programs, SAS, and STATA, while IBD information was generated from SOLAR. Allele frequencies were rescaled when they did not sum to 1 exactly.

### The analysis

The analysis was limited to microsatellite markers on chromosomes 4 and 7 based on prior publications [5]. For comparison, we also used STATA, which allows for frailty with a gamma distribution and two-level analysis. Often IBD matrices from SOLAR were taken by *kinship *to be non-positive definite (possibly due to rounding errors); therefore, we perturbed the IBD matrices with an identity matrix with small variance (0.01). Terry Therneau has indicated that this was not the case with SIMWALK2. However, SOLAR was unable to read outputs from SIMWALK2.89. Because *kinship *uses SOLAR output by default, the SIMWALK 2.89 results were not used. GENEHUNTER was used to obtain marker informativeness. Because each analysis can be viewed as multiple tests to identify a susceptibility gene on the two chromosomes, we computed false-discovery rates using SAS PROC MULTTEST.

## Results

There were 266 individuals with no information and 643 (436 men, 207 women) with alcohol dependence, the mean (SD) ages of onset being 22.8 (9.2) and 21.6 (8.9). More men than women were alcohol dependent and smokers were more likely to be alcohol dependent. The default gamma frailty model of mean one in STATA with sex gave variance (SE) of 0.063 (0.034) and *χ*^2 ^= 5.11, *p *= 0.012 according to a 50:50 mixture of  and , comparable to a similar model from *coxph *with a variance estimate of 0.065, *p *< 0.0001. Simple random effects model of family membership by *coxme *gave a variance estimate of 0.078.

However, it is more appropriate to use a correlated frailty model with the kinship matrix. This led to a variance estimate of 0.22 and integrated  = 5.49 with *p *= 0.019 compared to the log-likelihood -3534.66 without using kinship information. D4S1645 (near GABRB1) gave the largest contribution to the log-likelihood and highest variance (0.221) on chromosome 4. In comparison, D7S509 had the largest variance (0.238) and largest contribution to the log-likelihood on chromosome 7. The false-discovery rates were 0.044 and 0.030, respectively. When fixing the variance associated with the kinship matrix at the value of 0.22, D4S1645 showed the biggest difference in likelihoods. Due possibly to the similarity between structures of the kinship and IBD for random effects, most microsatellite markers and kinship relationship seemed to be strongly correlated, except markers D4S1645, D7S629, and D7S1830. There did not appear to be a link with marker informativeness (information contents of 58.9%, 53.6%, 61.6% according to GENEHUNTER). These results are shown in Table 1, where the *χ*^2 ^for the integrated log-likelihood is listed. Similar results were obtained when including sex as a covariate (data not shown), with the regression coefficient (SE) across loci in the range of 0.21~0.23 (0.09~0.10) and *p*-value in the range of 0.017~0.038; however, the model-fitting was greatly improved, indicating the importance of covariates in characterizing age of onset. The false-discovery rates were 0.065 ( = 0.22) and 0.093 ( and  are free), respectively.

## Discussion

The framework of Therneau [2] can integrate information from several sources: the affection status and age of onset, the familial relationship, genetic information, and covariates. The Cox model is familiar to researchers and accommodates counting process [start, stop] notation and time-dependent covariates, alternative time scales, and multiple events/subject data. Gaussian random effects allow for efficient analysis of large genetic correlations. Genetic markers can also be used via a function called *lmekin *(linear mixed models with kinship) for the analysis of quantitative traits in a way similar to SOLAR. The extended Cox model and the linear mixed model are flexible in assessing the relative magnitude of genetic and environmental influences, including multiple genes. Additional functions include kinship calculation, use of external IBD matrices and sparse matrices to analyze large, extended families, and pedigree-drawing for a single or a set of pedigrees. The package is more comprehensive than SAS and STATA in dealing with frailty, and the port to R makes it freely available to many platforms and represents a growing trend of integration of statistical genetics into the mainstream statistical computing. For instance, some data preparations done by C programs turned out to be much easier with the function *read.fortran *as available from R 2.0.0. The model is a natural generalization of the longitudinal model incorporating subject-specific random effects as given in [9]. It is also a hybrid between the model for quantitative trait locus linkage using times, and the model for discrete trait linkage using events. Unlike the parametric (LOD score) and nonparametric linkage analysis, which are well established [10], the Cox model approach is relatively new and requires further development.

D4S1645 appears to be promising but requires further investigation, e.g., the use of IBD information from SIMWALK2 and the exploration of the rapid analysis of many markers. It is possible to use information on the initiation of alcohol drinking and environmental factors such as smoking or social class when available. Ideally, tightly linked markers can be used in the models for association analysis and comparison made with other models [1,3,4].

## Abbreviations

COGA: Collaborative Study on the Genetics of Alcoholism

GAW14: Genetic Analysis Workshop 14

IBD: Identity by descent

## Figures and Tables

**Table 1:** The integrated *χ*^2^, *p*-value, and variances for kinship () and IBD () matrices for markers with successful optimization

	= 0.22			and are free			
		
Name		*p*			*p*		
D4S2366	4.79	0.091	0.026	5.48	0.064	0.415	1 × 10^-6^
D4S2639	5.00	0.082	0.057	5.48	0.064	0.414	1 × 10^-6^
D4S2382	4.91	0.086	0.048	5.48	0.064	0.415	1 × 10^-6^
GABRB1	5.37	0.068	0.088	5.48	0.064	0.415	1 × 10^-6^
D4S1645	6.03	0.049	**0.121^a^**	6.08	0.048	**0.110**	**0.165**
D4S1558	4.77	0.092	0.022	5.48	0.064	0.415	1 × 10^-6^
D4S1559	5.96	0.051	0.113	6.24	0.044	1 × 10^-6^	0.202
ADH3	5.06	0.080	0.062	5.48	0.065	0.415	1 × 10^-6^
FABP2	5.97	0.050	0.117	6.12	0.047	0.001	0.206
D4S1625	5.63	0.060	0.101	5.63	0.060	0.232	0.096
D4S1629	6.06	0.048	0.102	6.29	0.043	1 × 10^-6^	0.175
D4S1626	4.89	0.087	0.039	5.48	0.065	0.415	1 × 10^-6^
D4S2374	5.42	0.066	0.090	5.49	0.064	0.372	0.023
D4S171	4.78	0.092	0.022	5.48	0.064	0.415	1 × 10^-6^
D4S1652	6.70	0.035	0.123	7.43	0.024	1 × 10^-6^	0.192
							
D7S513	5.03	0.081	0.054	5.48	0.064	0.414	1 × 10^-6^
D7S1802	5.73	0.057	0.099	5.79	0.055	0.056	0.165
D7S629	5.80	0.055	0.101	5.84	0.054	**0.104**	**0.146**
D7S1838	5.40	0.067	0.083	5.49	0.064	0.376	0.020
NPY2	6.18	0.045	0.134	6.65	0.036	1 × 10^-6^	0.229
D7S817	5.01	0.082	0.054	5.48	0.064	0.414	1 × 10^-6^
D7S2846	6.17	0.046	0.132	6.48	0.039	1 × 10^-6^	0.225
D7S521	6.41	0.041	0.137	6.88	0.032	1 × 10^-6^	0.223
D7S691	4.82	0.090	0.028	5.48	0.064	0.415	1 × 10^-6^
D7S478	6.02	0.049	0.121	6.30	0.043	1 × 10^-6^	0.213
D7S679	5.32	0.070	0.081	5.48	0.064	0.415	1 × 10^-6^
D7S665	5.41	0.067	0.081	5.48	0.064	0.415	1 × 10^-6^
D7S1830	5.59	0.061	0.101	5.60	0.061	**0.150**	**0.133**
D7S3046	6.26	0.044	0.131	6.65	0.036	1 × 10^-6^	0.220
D7S1870	6.36	0.042	0.132	6.83	0.033	1 × 10^-6^	0.217
D7S1797	4.85	0.088	0.035	5.48	0.064	0.415	1 × 10^-6^
D7S820	5.79	0.055	0.113	5.90	0.052	1 × 10^-6^	0.212
D7S1799	6.07	0.048	0.125	6.40	0.041	1 × 10^-6^	0.217
D7S1817	4.75	0.093	0.013	5.48	0.064	0.415	1 × 10^-6^
D7S509	5.86	0.053	0.123	6.02	0.049	1 × 10^-6^	0.228

^a ^Bold text indicates large values for both variances.
